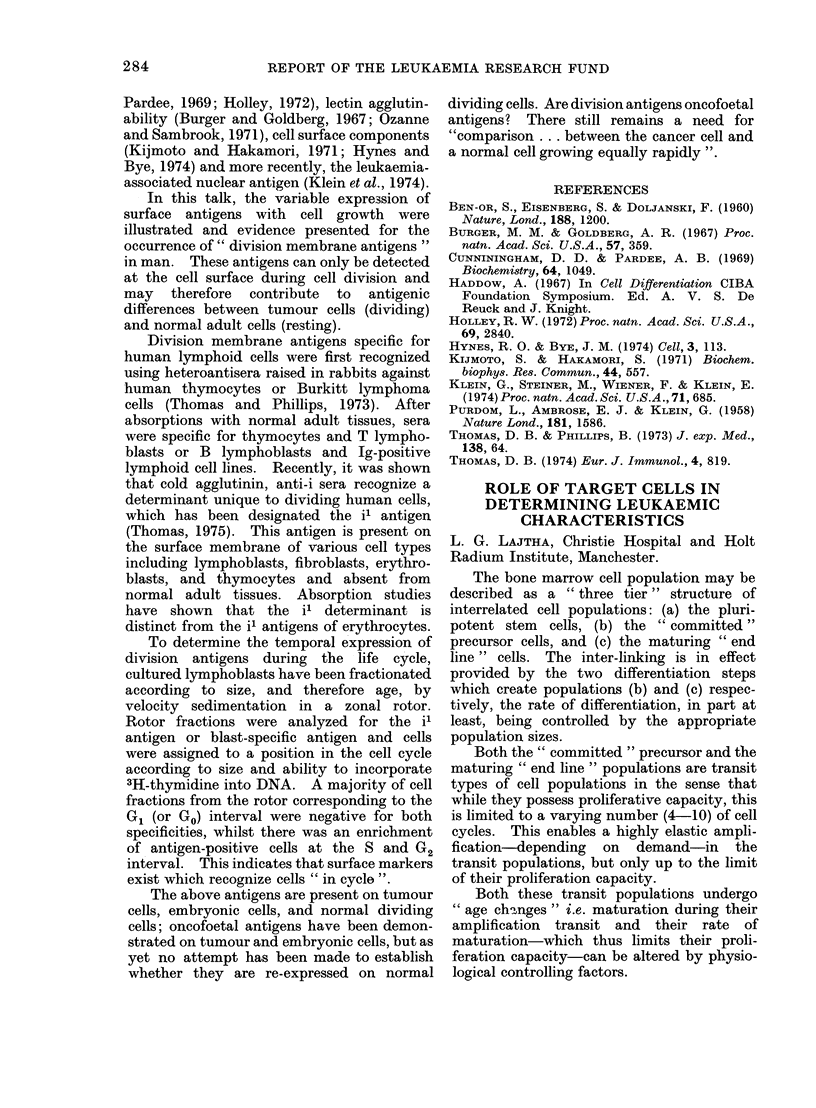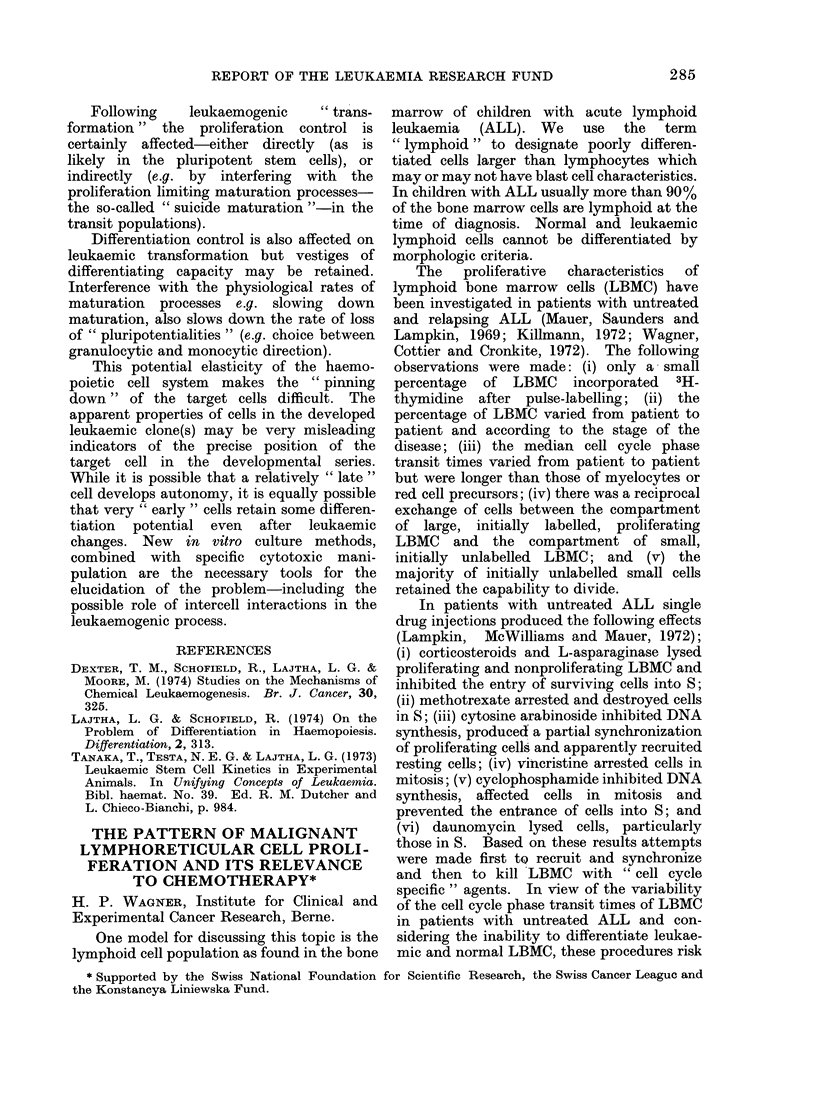# Proceedings: Role of target cells in determining leukaemic characteristics.

**DOI:** 10.1038/bjc.1975.222

**Published:** 1975-08

**Authors:** L. G. Lajtha


REFERENCES

### ROLE OF TARGET CELLS IN DETERMINING LEUKAEMIC CHARACTERISTICS

L. G. Lajtha, Christie Hospital and Holt Radium Institute, Manchester.

The bone marrow cell population may be described as a " three tier " structure of interrelated cell populations: (a) the pluripotent stem cells, (b) the " committed " precursor cells, and (c) the maturing " end line " cells. The inter-linking is in effect provided by the two differentiation steps which create populations (b) and (c) respectively, the rate of differentiation, in part at least, being controlled by the appropriate population sizes.

Both the " committed " precursor and the maturing " end line " populations are transit types of cell populations in the sense that while they possess proliferative capacity, this is limited to a varying number (4—10) of cell cycles. This enables a highly elastic amplification—depending on demand—in the transit populations, but only up to the limit of their proliferation capacity.

Both these transit populations undergo " age changes " *i.e.* maturation during their amplification transit and their rate of maturation—which thus limits their proliferation capacity—can be altered by physiological controlling factors.

Following leukaemogenic " transformation " the proliferation control is certainly affected—either directly (as is likely in the pluripotent stem cells), or indirectly (*e.g.* by interfering with the proliferation limiting maturation processes—the so-called " suicide maturation "—in the transit populations).

Differentiation control is also affected on leukaemic transformation but vestiges of differentiating capacity may be retained. Interference with the physiological rates of maturation processes *e.g.* slowing down maturation, also slows down the rate of loss of " pluripotentialities " (*e.g.* choice between granulocytic and monocytic direction).

This potential elasticity of the haemopoietic cell system makes the " pinning down " of the target cells difficult. The apparent properties of cells in the developed leukaemic clone(s) may be very misleading indicators of the precise position of the target cell in the developmental series. While it is possible that a relatively " late " cell develops autonomy, it is equally possible that very " early " cells retain some differentiation potential even after leukaemic changes. New *in vitro* culture methods, combined with specific cytotoxic manipulation are the necessary tools for the elucidation of the problem—including the possible role of intercell interactions in the leukaemogenic process.

REFERENCES

DEXTER, T. M., SCHOFIELD, R., LAJTHA, L. G. & MOORE, M. (1974) Studies on the Mechanisms of Chemical Leukaemogenesis. *Br. J. Cancer*, **30**, 325.

LAJTHA, L. G. & SCHOFIELD, R. (1974) On the Problem of Differentiation in Haemopoiesis. *Differentiation*, **2**, 313.

TANAKA, T., TESTA, N. E. G. & LAJTHA, L. G. (1973) Leukaemic Stem Cell Kinetics in Experimental Animals. In *Unifying Concepts of Leukaemia*. Bibl. haemat. No. 39. Ed. R. M. Dutcher and L. Chieco-Bianchi, p. 984.